# Using the Ghana malaria indicator survey to understand the difference between female and male-headed households and their prevention and testing for malaria among children under 5

**DOI:** 10.1186/s12936-022-04135-4

**Published:** 2022-04-02

**Authors:** Daniel Iddrisu, Cheryl A. Moyer

**Affiliations:** 1 International and Regional Studies, University of Michigan, Ann Arbor, MI USA; 2 Department of Learning Health Sciences, University of Michigan Medical School, Ann Arbor, MI USA; 3 Department of Obstetrics and Gynecology, University of Michigan Medical School, Ann Arbor, MI USA

**Keywords:** Malaria, Health disparities, Female-headed households, Africa, Low- and middle-income countries

## Abstract

**Background:**

Globally, 94% of malaria deaths occur in sub-Saharan Africa, and children under age 5 account for 70% of malaria-related mortality in the region. This study sought to examine differences between female-headed households (FHHs) and male-headed households (MHHs) with regard to malaria prevention and testing among children under age 5 (U5) in Ghana.

**Methods:**

This cross-sectional study used publicly available data from the 2019 Ghana Malaria Indicator Survey (GMIS). Frequencies and descriptive statistics were calculated for all key variables. Bivariate analyses comparing FHHs and MHHs were conducted using t tests and Chi-square analysis. A P value of 0.05 was taken for statistical significance.

**Results:**

Five thousand one hundred and eighty one household were identified, of which 1938 (37.4%) were female-headed and 3243 (62.6%) were male-headed. 51.7% of FHHs included a child U5, whereas 67.8% of MHHs included a child U5. MHHs were significantly more likely to own an ITN than FHHs (83.1% vs. 78.3%, P < 0.001), whereas FHHs were more likely to report taking malaria prevention steps such as spraying the house with insecticide, filling in stagnant puddles, and keeping surroundings clear (all significant at P < 0.001). U5 children in MHHs were more likely to sleep under a bed net the night preceding the survey (51.0%) than U5 children in FHHs (44.8%), although the finding was not statistically significant. The rates of fevers in the previous two weeks among children U5 were similar across MHH and FHH (24.2% vs. 22.3%), and the rates of testing for malaria among those who experienced a febrile episode were also similar across MHHs and FHHs (39.0% vs. 41.3%). Of those tested, the percentage of U5 children who tested positive for malaria was also similar across MHHs and FHHs (63.9% vs. 63.0%).

**Conclusions:**

Both FHHs and MHHs in Ghana make a concerted effort to prevent and test for malaria among children U5 in their households. Despite differences in malaria prevention strategies, there were no significant difference in febrile episodes, malaria testing, and rates of positivity, suggesting that malaria prevention is challenging for all households in Ghana. In the face of a newly-developed malaria vaccine, future research is warranted to ensure adequate uptake across all households.

## Background

Malaria continues to be a significant problem in sub-Saharan Africa (SSA), despite substantial efforts focused on prevention, screening, and treatment. In 2018, there were 228 million reported cases of malaria and 405,000 deaths in SSA [1]. The World Health Organization (WHO) indicated that 94% of malaria deaths occurred in sub-Saharan Africa [2].

Although malaria affects all age groups, it is specifically problematic for children under age five (U5) [3]. Though the prevalence of malaria among children under age five in SSA dropped by 18% between 2000 and 2016, children still constitute 70% of malaria-related deaths in SSA [4].

In Ghana, malaria cases account for 10.4 million outpatient visits per year, 4.2% of which are children U5 [5]. This percentage looks small but requires attention since malaria infection is said to claim the live of one child under 5 years of age in every 2 mins in the country [6]. By the first quarter of 2020, Ghana had recorded more than 1 million malaria cases, with 54 children under the age of 5 losing their lives to malaria [7].

With the persistent prevalence of malaria among children U5 in Ghana, the government and other international agencies have given critical attention to strategies that will help prevent malaria in the country [8]. Malaria prevention in Ghana is often characterized by the use of insecticide-treated bed nets (ITNs), mosquito repellents, filling in stagnant waters (puddles), among others. Public health education and provision of financial support is granted to families and individuals to ensure that such malaria prevention strategies are well adopted and used [9].

Previous studies have suggested a strong correlation between the use of insecticide treated bed nets and the presence of children U5 in FHHs [10], but it is not known how FHHs compare to MHHs with regard to malaria prevention and testing. This study sought to accomplish three aims: (1) To compare the sociodemographic characteristics of female-headed households and male-headed households in Ghana; (2) To determine differences between FHHs and MHHs with regard to malaria preventive behaviors such as ownership and use of ITNs; and (3) To determine differences between FHHs and MHHs with regard to malaria symptoms, testing, and rates of positivity.

## Methods

This cross-sectional study was conducted using de-identified, publicly available data from the 2019 Malaria Indicator Survey in Ghana [11]. The Ghana Malaria Indicator Survey (GMIS) is part of the Demographic and Health Survey (DHS) program, which is a program that collects, analyses and disseminates nationally representative data on populations, health, HIV, and nutrition across more than 90 countries.

The GMIS was conducted in Ghana, a nation in west Africa located along the Gulf of Guinea, a few degrees north of the equator. The geography of Ghana is variable, ranging from coastal plains in the south to dry, arid regions in the north. The economy of Ghana is agriculturally driven, with over 60% of the population of the country working in agriculture and many families reliant upon subsistence farming.

The GMIS reflects nation-wide sampling of households, and it assessed demographic and health-related variables [12]. While the GMIS includes interviews across a total of 5799 households, a total of 5181 women aged 15–49 successfully completed individual interviews [11]. The data analysed in this study reflects data from 5181 individual interviews with women. Key variables used in this study included: household leadership (female-headed households, male-headed households), demographics of the household head (including age, education, rural/urban residence), presence of children under age 5 in the household, ownership of insecticide-treated bed nets (ITNs), children under 5 sleeping under ITN in the night before the survey, febrile episodes among children under 5, malaria testing among children under 5, malaria results among children under age 5.

The GMIS was conducted in 2019 using 52 field workers who went through training from 2nd to 21st of September 2019. Field workers visited randomly selected households within the then 10 enumeration regions reflective of Ghana’s 2010 Population Census. Field workers administered the GMIS survey to all women aged 15–49 who were either permanent residents of the selected households or visitors who stayed in the household the night before the survey [13]. Malaria testing was conducted using Rapid Diagnostic Testing with blood samples taken from the finger or heel prick and test results were confirmed using microscopy at the Nation Public Health and Reference Laboratory. Treatment was sought immediately for the children whose test results came out positive for malaria.

## Data analysis

Data files were stratified for FHHs and MHHs to achieve the results that differentiate between household headships. R and Stata 16.0 analytical programs were used to calculate frequencies and descriptive statistics for all key variables. Bivariate analyses comparing female-headed households and male-headed households were conducted using t tests and Chi-square analysis. A P value of 0.05 was taken for statistical significance.

## Results

Among the 5181 interviews completed in the GMIS, 62.6% (N = 3243) of respondents reported living in male-headed households and 37.4% (N = 1938) reported living in female-headed households (Table 1). In comparing female-headed households (FHHs) and male-headed households (MHHs) (per Aim 1), male-headed households had more individuals living in the household (P < 0.001) and more children under age 5 living in the household (P < 0.001). FHHs were more likely to be located in an urban area, female household heads were likely to be better educated than male household heads, and FHHs had higher overall wealth index scores than MHHs (P < 0.001).

**Table 1:** Household demographics from 2019 Ghana Malaria Indicator Survey, unweighted (N = 5181)

Variable	Female-headed households (N = 1938)	Male-headed households (N = 3243)	P value
Mean age (± SD)	29.4 (± 9.8)	29.9 (9.5)	0.08
Mean # of individuals in household	4.5 (± 2.5)	6.4 (± 3.5)	< 0.001
# of children 5 and under in household	0.8 (± 0.95)	1.2 (± 1.2)	< 0.001
% in urban residence	58.5 (1134)	40.3 (1306)	< 0.001
Highest level of education	3.2 (1.4)	2.9 (1.4)	< 0.001
No education	14.3 (277)	26.4 (856)	
Primary	16.4 (318)	19.8 (642)	
Secondary	60.3 (1168)	48.2 (1564)	
Higher	9.0 (175)	5.6 (181)	
Wealth index combined	3.1 (1.3)	2.7 (1.5)	< 0.001
Poorest	14.4 (279)	32.3 (1047)	
Poorer	19.8 (383)	17.5 (567)	
Middle	25.4 (493)	16.7 (542)	
Richer	19.8 (383)	16.2 (526)	
Richest	20.6 (400)	17.3 (561)	

**Fig. 1:**
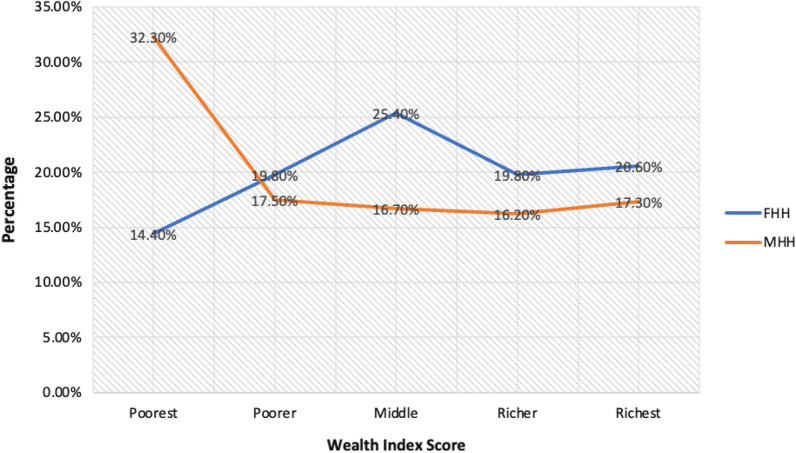
A comparison of wealth across female-headed and male-headed households, 2019 GMIS

Figure 1 illustrates the differences in wealth between female-headed and male-headed households. This figure reflects a cumulative wealth index score that was divided into quintiles and labeled with the categories of poorest, poorer, middle, rich and richest. Notably, MHHs are much more likely to fall in the ‘poorest’ category than female-headed households, with 32.3% of male-headed households labeled as ‘poorest’ in comparison to only 14.4% of female-headed households (Fig. 1).

**Fig. 2:**
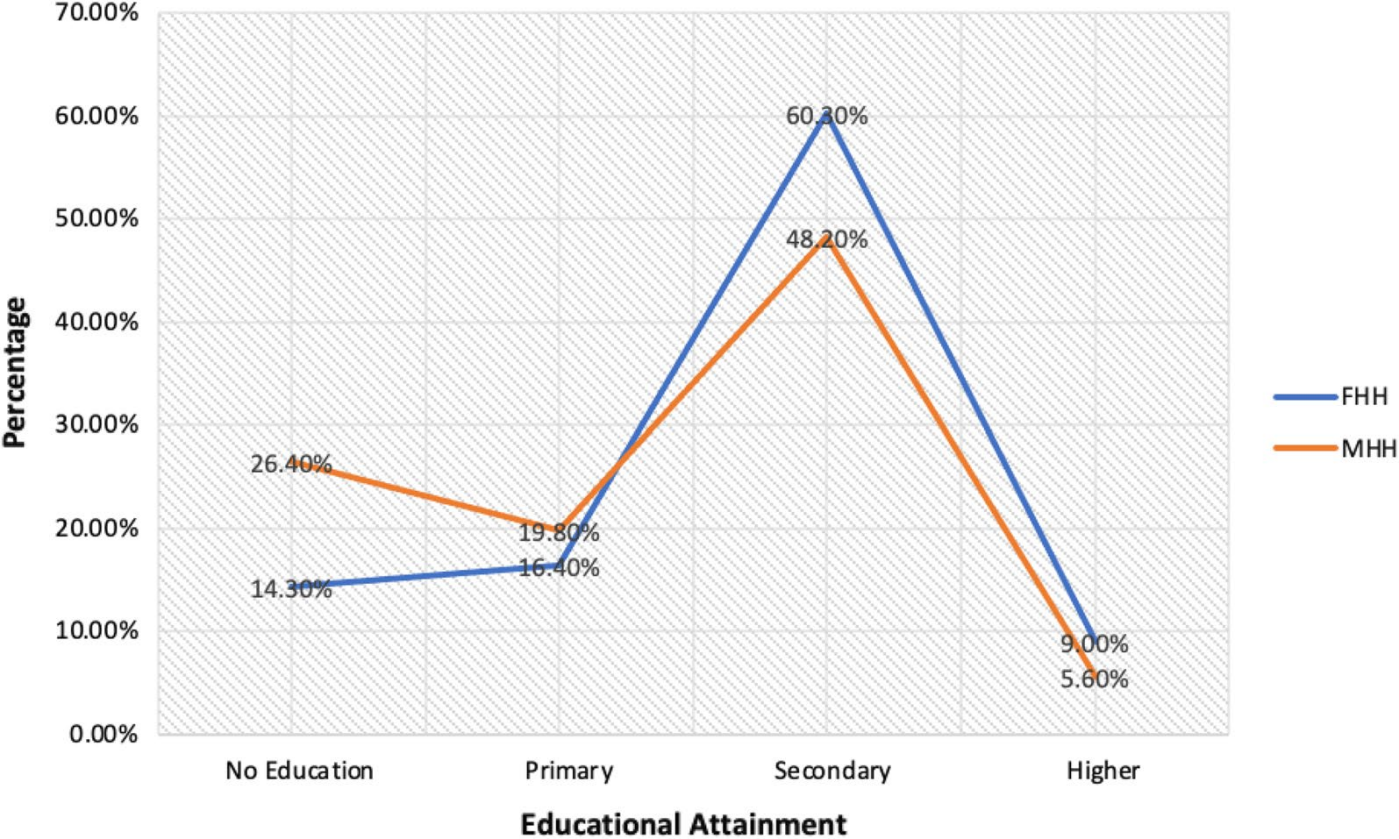
Differences in educational attainment between female-headed and male-headed households, 2019 GMIS

Figure 2 is an illustration of the differences in educational attainment across FHHs and MHHs. The educational attainment between FHHs and MHHs are assessed based on those who had no education, those who had up to primary education, those with secondary education and higher. Overall, women in male-headed households were less educated than women in female-headed households, with 26.4% of women in male-headed households having no education, compared to 14.3% of FHH (Fig. 2).

In terms of Aim 2, determining the differences between FHHs and MHHs with regard to malaria preventive behaviors such as ownership and use of ITNs, and Aim 3, determining the differences between FHHs and MHHs with regard to malaria symptoms, testing, and rates of positivity, Tables 2 and 3 illustrate the malaria-related variables that were examined. MHHs were more likely to own an insecticide treated bed net (ITN) and were slightly more likely to report that all children under age 5 slept under an ITN the previous night, although this was not statistically significant. There was no difference between MHHs and FHHs with regard to the percentage whose children under 5 had a fever in the previous two weeks, were tested for malaria, or who tested positive for malaria (Table 2).

**Table 2:** Malaria-related variables from 2019 Ghana Malaria Indicator Survey, unweighted

Variable	Female-headed households (N = 1938) % (N)	Male-headed households (N = 3243) % (N)	P value
Owns an ITN	78.3 (1518)	83.07 (2694)	< 0.001
Has child under 5 living in household	51.7 (1002)	67.8 (2200)	
All children under 5 slept under an ITN last night	44.8 (449)	51.0 (1122)	0.45
Children under 5 with fever in the last 2 weeks before the survey	22.3 (223)	24.2 (533)	0.97
% of children under 5 with a fever who had a blood test for malaria	41.3 (92)	39.0 (208)	0.40
% of children tested who tested positive for malaria	63.0 (58)	63.9 (133)	0.594

**Table 3:** Malaria preventive behaviors by household type, 2019 GMIS

Malaria prevention variable	Female-headed households (N = 1938)	Male-headed households (N = 3243)	P value
Take malaria prevention medication	4.13 (80)	3.45 (112)	0.214
Sleep under mosquito net	39.53 (766)	38.05 (1234)	0.292
Used mosquito repellent	12.33 (239)	11.35 (368)	0.286
Spray house with insecticide	20.33 (394)	14.99 (486)	< 0.001
Fill in stagnant waters (puddles)	26.21 (508)	20.84 (676)	< 0.001
Keep surrounding clear	57.43 (1113)	49.21 (1596)	< 0.001
Put mosquito screen on windows	0.88 (17)	0.59 (19)	0.222
Other	5.16 (100)	4.50 (146)	0.281
Don’t know	1.55 (30)	3.21 (104)	< 0.001

Table 3 illustrates malaria prevention related variables adopted by both FHHs and MHHs. Taking malaria prevention medication, sleeping under a mosquito net, and using mosquito repellent are equally likely across FHHs and MHHs, with no statistically significant difference. However, FHHs are significantly more likely to spray their households with insecticides and fill in stagnant waters (puddles) as well as keep surrounding clean compared to MHHs. This is statistically significant at P < 0.001. A slightly higher percentage of MHHs do not know any malaria prevention ways compared to the FHHs (Table 3).

**Fig. 3:**
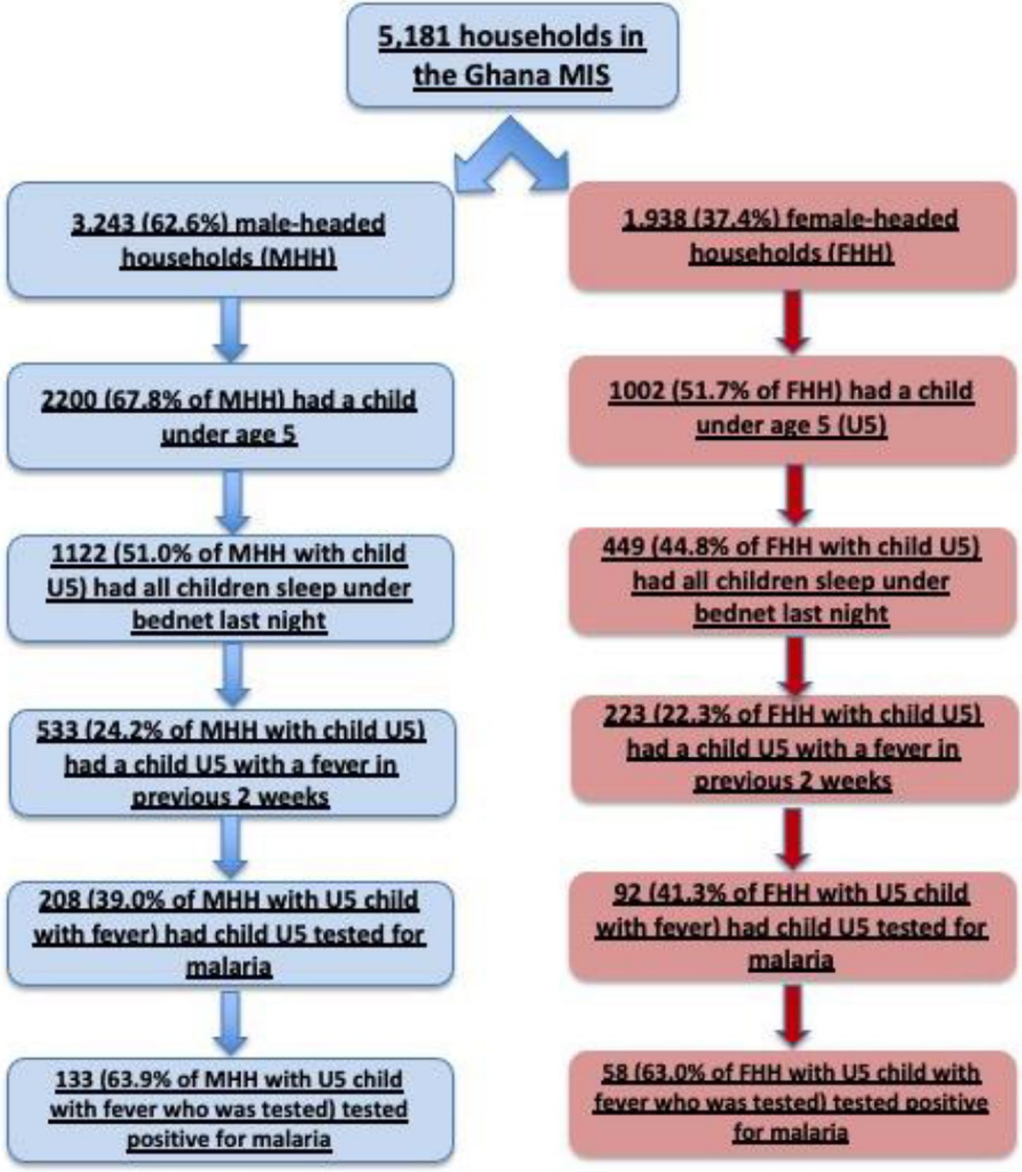
Ghana 2019 Malaria Indicator Survey Summary of Household Comparisons

Figure 3 illustrates the summary of the differences between household types with regard to the percentage who have children under age 5 in the household, percentage whose children under age 5 slept under a bed net the previous night, percentage whose U5 child had a fever in the previous 2 weeks, and percentage whose child with a fever was tested for malaria, and the percentage of those whose child was tested were indeed positive for malaria (Fig. 3).

## Discussion

While female-headed households with children U5 report a greater likelihood of malaria prevention strategies such as spraying the house with insecticide, filling in stagnant puddles around their household, and keeping surrounding areas clear, male-headed households with children U5 were more likely to own insecticide-treated bed nets. Nonetheless, there was no significant difference between FHHs and MHHs in terms of the number of U5 children with fevers in the previous 2 weeks, the number who sought malaria testing as a result, or the number who tested positive for malaria. These findings run counter to significant sociodemographic differences across MHHs and FHHs, with female household heads having greater education, higher wealth, and a higher likelihood of living in an urban area—all factors that one might assume would predispose FHHs to have lower rates of fevers and malaria among children U5.

The findings confirm other studies conducted in Ghana that compared FHHs and MHHs using the consumption expenditure approach, particularly data from the Ghana Living Standards Survey (GLSS) that suggests that FHHs are wealthier than MHHs [11]. Yet the consumption expenditure approach focuses solely on economic indicators (income, consumption, expenditures), which may overlook other types of important assets that differ by household type [14]. A different approach to comparing FHHs and MHHs (the livelihoods approach) involves examining the multidimensional nature of living conditions, including access to assets, social networks, and capabilities (education, skills, and health) [14]. Using this approach, [15] found that FHHs do not have access to key assets and, therefore, are not as well off as MHHs.

These two different approaches to assessing differences in poverty status across household type raise a fundamental question: how does one define ‘better off’? In terms of asset accumulation, women in MHHs often play critical roles by taking care of the children and contributing to the upkeep and maintenance of the household, giving the male household head time to work and acquire more asserts for the household [16]. This cannot be said in the case of FHHs, where the female head is often on her own to earn money for the household as well as take care of any children in it [17]. Yet the authors also know that social networks among women can be very strong, and social networks can play an important role in providing non-financial, non-asset-oriented assistance, such as sharing food, childcare, and other domestic responsibilities.

It is also important to note that female-headed households may not all look the same. While traditionally defined as “a woman in charge of managing the family as a result of divorce, separation, immigration, or widowhood” [18], FHHs may range from a young mother with small children at home to an older woman living with several grown children. Obviously, the assets and resources across such different types of living situations are likely to be extremely variable.

Traditionally, some of the factors that limit FHHs’ access to assets include tradition and role differentiation for women and men [19]. Yet role differentiation may prove helpful in the case of care seeking for illness or preventive care, as it is typically women who take such responsibility for themselves and their children. With regard to testing and treatment for malaria, the findings are consistent with other studies in Ghana showing that FHHs who were consistent in making hospital maternal visits during pregnancy continued to take their children to the hospital for check-ups and malaria related tests after their pregnancies [20].

## Significance of these findings

This study has several important implications. First, MHHs and FHHs engaged in different types of malaria-prevention behaviors, yet they ultimately did not affect the proportion of U5 children in either type of household who experienced febrile episodes, were tested for malaria, or tested positive for malaria. This raises questions about the utility of the malaria prevention methods being promoted altogether (targeting both FHHs and MHHs)—or whether additional implementation science research is warranted to determine if the interventions are being conducted as intended.

Though there are variations between FHHs and MHHs in terms of malaria prevention and control, both household groups appear to be underperforming. These findings also provide support for rapid and widespread deployment of the newly developed malaria vaccine, RTS,S/AS01_E,_ even though it has shown to prevent severe infections by only 30% (RTS,S Clinical Trials Partnership [21]. While additional trials have shown that combining the vaccine with seasonal malaria prophylaxis can significantly boost its protective effects [22], widespread adoption and uptake will be necessary across both male- and female-headed households if U5 malaria death rates are to be reduced.

This study also showed that MHHs and FHHs differed significantly in terms of wealth, education, rural/urban status, number of children U5, and ownership of bed nets. Yet none of these sociodemographic variables seemed to matter in terms of malaria infection. This is an important finding because efforts to target low-income, rural Ghanaians may miss higher-income, urban families who are equally at risk.

## Limitations of this study

This study has several limitations. First, the Ghana Malaria Indicator Survey (GMIS) is based on self-reported data, which may be subject to both recall bias and social-desirability bias. In addition, malaria is seasonal, and thus the timing of the GMIS may affect its results. Despite an initial household sample size of more than 5000 households, the number of households with children under 5 who had a fever within the previous 2 weeks and who sought a malaria test for the child is relatively small. It is possible there was insufficient power to detect a statistically significant difference between MHHs and FHHs in this exploratory analysis.

## Conclusions

Ghana Malaria Indicator Survey Data suggest that both FHHs and MHHs in Ghana make a concerted effort to prevent and test for malaria among children under 5 in their households. While there were differences between FHHs and MHHs in terms of the malaria prevention strategies they employ, there were no significant differences in febrile episodes, malaria testing, and rates of positivity, suggesting that the issues surrounding malaria prevention, infection, and treatment are universally challenging. Nonetheless, they are particularly challenging for FHHs in Ghana because of two issues: government public health policies that aim to distribute free bed nets may not be reaching FHHs, as indicated by [23], and the limited financial resources of FHHs may hamper the purchase of malaria prevention equipment. In the face of a newly-developed malaria vaccine, future research is warranted to ensure adequate uptake across all households.

## Data Availability

All data used in this study are available through the Demographic Health Survey Program, dhsprogram.com/data.
